# Evaluating the Necessity of a Control Treatment for Assessing Salt Tolerance in Wheat Genotypes Based on Agro-Physiological Traits in Real-Field Conditions

**DOI:** 10.3390/plants14162488

**Published:** 2025-08-11

**Authors:** Salah El-Hendawy, Muhammad Usman Tahir, Yuncai Hu, Nasser Al-Suhaibani

**Affiliations:** 1 Department of Plant Production, College of Food and Agriculture Sciences, King Saud University, P.O. Box 2460, Riyadh 11451, Saudi Arabia; 2 Precision Agriculture Laboratory, School of Life Sciences, Technical University of Munich, 85354 Freising, Germany

**Keywords:** canopy temperature, chlorophyll contents, Fv/Fm, heatmap clustering, ionic contents, grain yield, stress tolerance indices, salinity stress

## Abstract

Evaluating salt tolerance based on agro-physiological traits is resource-intensive when testing numerous genotypes under both control and saline conditions. Focusing specifically on stress conditions may streamlines the process while effectively revealing the physiological mechanisms underlying salt tolerance in genotypes. This study investigated whether control treatments are necessary for accurate salt tolerance assessment by analyzing 22 wheat genotypes under real field conditions with control and 150 mM NaCl salinity. Genotypes were grouped based on absolute trait values of ionic and agro-physiological traits under normal or salinity stress conditions separately, as well as stress tolerance indices (STIs) that consider the genotypes’ performance under stress compared to non-stress conditions. Heatmap clustering of ionic, physiological, or growth and yield traits under salinity stress successfully differentiated between salt-tolerant (Sakha 93) and sensitive (Sakha 61) genotypes. In contrast, the heatmap of ionic and physiological traits under control conditions or STIs of ionic and growth and yield traits failed to distinguish between the two genotypes. When categorized based on control-condition values or STIs, the Sakha 93 group performed similarly or worse than the Sakha 61 group. However, under salinity stress, the Sakha 93 group consistently outperformed the Sakha 61 group. Salinity-stress trait values provided significant insights into the salt tolerance mechanisms of the tested genotypes, whereas control condition data offered no meaningful contribution to understanding salinity tolerance. In summary, assessing ionic and agro-physiological traits under salinity stress alone can accurately evaluate the salt tolerance of wheat genotypes in real field conditions, eliminating the necessity of determining them under control conditions. This method not only saves effort, time, and resources when evaluating the salt tolerance of a large number of genotypes, but also offers a reliable way to understand the mechanisms of salt tolerance through agro-physiological traits.

## 1. Introduction

Wheat (*Triticum aestivum* L.) is a crucial staple crop that contributes significantly to global food security. It provides 30% of the world’s population with 33% of their protein needs and 24% of their calorie intake [1]. To meet the needs of the growing global population, which is projected to reach 9.5 billion by 2050, wheat production must double by then [1]. However, wheat production faces major abiotic stress challenges, particularly salinity stress, which can reduce grain yield by over 60% due to wheat’s moderate tolerance [2,3]. This problem is projected to intensify with climate change through rising temperatures, higher evaporation, and lower rainfall, especially in arid and semiarid regions. Hence, immediate and affordable solutions are required to address salinity stress in wheat. Genetic enhancements can increase stress tolerance, leading to a potential 25% increase in productivity compared to unimproved varieties [4]. Offering salt-tolerant genotypes is the most sustainable and practical approach, eliminating the need for expensive methods such as gypsum application or soil leaching.

Assessing genetic diversity in salt tolerance across diverse wheat genotypes is the initial step in breeding programs, aiming to identify high-tolerance donors for incorporation into improved varieties. While this step does not require extensive experience, progress in improving the salt tolerance of wheat genotypes is still limited. As a result, only a few salt-tolerant genotypes have been released through breeding efforts so far, and fewer salt-tolerant wheat varieties have been implemented in practical applications. According to previous literature, this lack of progress is due to several reasons, including the following: (1) evaluating a diverse range of genotypes using various traits under both controlled and salt-stress conditions can be a costly, labor-intensive, and time-consuming process; (2) evaluations for salinity tolerance are usually conducted in greenhouses using hydroponics or sand soil as growing media, which may not fully mimic the intricate field conditions; (3) many studies that rely on traditional tools to evaluate salt tolerance may not accurately identify salt-tolerant genotypes, particularly when using the tools that assess genotype performance under salt stress conditions compared to normal conditions, such as stress tolerance indices (STIs); (4) a limited understanding of the complex mechanisms underlying salt tolerance; (5) the limited use of physiological traits as screening criteria for assessing salt tolerance in genotypes; and (6) the lack of using multiple traits simultaneously to facilitate ranking genotypes for salt tolerance [2,3,5,6,7,8,9,10,11,12].

The main sign of excessive salt levels in the root zone is the occurrence of ion toxicities and imbalances. Elevated root-zone salt levels lead to toxic Na^+^ accumulation in shoots while promoting competitive Na^+^ uptake over K^+^ and Ca^2+^ at root absorption sites, ultimately reducing the ratio of intercellular K^+^/Na^+^ and Ca^2+^/Na^+^ ratios. Therefore, previous research has demonstrated that salt-tolerant wheat genotypes typically can limit Na^+^ uptake while maintaining high intracellular K^+^/Na^+^ and Ca^2+^/Na^+^ ratios [12,13,14,15]. Consequently, the concentrations and ratios of these specific ions in shoots can serve as valuable screening criteria for plant breeders to distinguish between salt-tolerant and salt-sensitive genotypes and to identify genetic donors that can be integrated into breeding programs for salt tolerance. The question is whether it is adequate to measure the concentration of these elements solely under salinity conditions, or if they should also be measured under control conditions to accurately assess the salt tolerance of wheat genotypes. If evaluating the salt tolerance of genotypes can be effectively done by estimating these elements only under salinity conditions, it would save effort, money, and time when evaluating a large number of genotypes. Under non-saline conditions, the concentrations and ratios of Na^+^, K^+^, and Ca^2+^ in the shoots almost remain consistent among various wheat genotypes. However, under saline stress, there are notable variations in the concentrations and ratios of these elements among genotypes. Additionally, high salinity levels resulted in a significant 10–20-fold increase in Na^+^ concentrations in shoots and a 2–3-fold decrease in K^+^ and Ca^2+^ concentrations in shoots compared to the control treatment [12,14,16,17]. This underscores the significance of measuring the concentration of these elements specifically in saline conditions, rather than in normal conditions, when assessing the salinity tolerance of numerous wheat genotypes and understanding their tolerance mechanisms based on ion content. Therefore, one objective of this study is to validate our hypothesis that measuring ion contents solely under saline conditions could be adequate as screening criteria for accurately assessing the salt tolerance of different wheat genotypes.

High levels of salt in the root zone can also cause osmotic stress, which can impede the plant’s ability to absorb water from the soil. This can disturb the plant’s water balance and lead to stomatal closure. Consequently, the plant may experience lower relative water content (RWC) and transpiration rates, both of which are crucial for regulating canopy foliage temperature (CT). When stomata close as a result of osmotic or ionic stress, it causes a notable reduction in transpiration rate, leading to a substantial increase in CT [12,18,19]. The combined effects of osmotic and ionic stress can also result in damage to the photosynthetic apparatus by damaging photosynthetic pigments (reducing chlorophyll synthesis and/or increasing chlorophyll breakdown) or reducing the maximum efficiency of photosystem II (the ratio of variable to maximal fluorescence; F_v_/F_m_). Both stresses can also cause changes in the structure and function of chloroplasts, ultimately leading to a decrease in overall chlorophyll content (Chlt) and chlorophyll fluorescence [20,21]. As a result, a reduction in Chlt consistently resulted in decreased chlorophyll fluorescence, even at low NaCl concentrations [22]. In this context, the significant correlation and interaction among the abovementioned physiological traits, i.e., RWC, CT, Chlt, and Fv/Fm, under salinity stress conditions confirm the effectiveness of these physiological traits as screening criteria for assessing the salt tolerance of wheat genotypes. However, the question that remains is whether it is sufficient to measure these physiological traits only under saline conditions, or if they should also be measured under normal conditions to accurately assess the salt tolerance of wheat genotypes. If possible, this approach could result in savings in effort, time, and resources when evaluating a large number of genotypes.

Previous studies have demonstrated that the RWC of various field crops, including wheat, barely, and rice genotypes, decreased significantly under salinity stress conditions compared to the control, and no significant variations were found between genotypes under control treatment [22,23,24]. Regarding Chlt, studies have shown that genotypes with the ability to maintain low Na^+^ content in leaves under salinity stress conditions showed high Chlt levels, while genotypes that accumulated high Na^+^ content in leaves experienced a significant decrease in Chlt [25,26]. Furthermore, some wheat genotypes did not show a significant reduction in Chlorophyll a, b, and Chlt content under salinity stress conditions compared to the control treatment, while others did exhibit a notable decrease [17]. Typically, Fv/Fm values fall between 0.76 and 0.85 under normal conditions. If the value drops below this range, it indicates the potential compromise or inactivity of specific PSII reaction centers, which is often observed in salt-sensitive genotypes under salinity stress conditions [20,27]. Stomatal closure is also a typical response to salinity stress, leading to reduced stomatal conductance and transpiration rates, reducing canopy cooling. This decrease in cooling causes an increase in CT [18,19]. Considering all of the foregoing information, measuring RWC, CT, Chlt, and Fv/Fm under salinity stress conditions may be a sufficient and accurate tool for assessing the salt tolerance of different wheat genotypes, without the necessity of measuring them under normal conditions.

Under salinity stress conditions, any disturbance or alteration in physiological pathways leads to significant changes in various morphological characteristics at the plant’s overall level. These changes can be observed at different plant growth stages. Therefore, combining agronomic traits with physiological traits can enhance the evaluation of salt tolerance in genotypes. For instance, salinity stress caused a notable decrease in photosynthetic rate due to stomatal and/or non-stomatal factors [28]. This led to a reduction in plant growth and yield due to the limited availability of photosynthetic products required for cell division and elongation as well as their transport from source to sink. Therefore, several agronomic traits associated with plant growth and yield, such as leaf area (LA), plant dry weight (PDW), grain yield (GY), and their components, are notably decreased by salinity stress. However, the extent of reduction in these traits due to salinity stress differs among wheat genotypes, with salt-tolerant genotypes experiencing less reduction compared to salt-sensitive ones [15,29,30,31]. These findings emphasize the significance of agronomic traits as a reliable indicator of wheat genotype performance under salinity stress conditions, as well as their level of salt tolerance. Therefore, these traits can be used as an applicable valid screening criterion to assess the salt tolerance of different wheat genotypes. However, the question that still needs to be answered is whether it is sufficient to record these agronomic traits only under salinity stress conditions, or if these traits should also be recorded under non-saline conditions to accurately assess the salt tolerance of wheat genotypes.

Plant breeders in a breeding program always target select genotypes that have genetic adaptability to tolerate salinity stress and demonstrate exemplary performance in terms of agro-physiological traits under both stressed and non-stressed conditions. To achieve this target, different stress tolerance indices (STIs) such as the stress tolerance index (STI), mean productivity index (MPI), and relative efficiency index (REI) are utilized to assess genotype performance under both stress and non-stress conditions simultaneously [10,13,32]. The STI can identify genotypes performing well under stress conditions while showing high stress tolerance. Therefore, this index is valuable for distinguishing between salt-tolerant and salt-sensitive genotypes [33,34]. On the other hand, the MPI can identify genotypes that perform well under both stress and non-stress conditions but may not accurately identify genotypes with high stress tolerance, even if they perform poorly under stress conditions [35]. To calculate the different STIs based on agro-physiological traits, these traits should be recorded for control and salinity stress conditions. However, when assessing various genotypes for their performance, especially in terms of agronomic traits under stress and non-stress conditions, they can be categorized into four groups: genotypes that perform poorly in both conditions, genotypes that perform well in both conditions, genotypes that excel in only non-stress conditions, and genotypes that excel in only stress conditions. This indicates that certain STIs may not effectively evaluate the salt tolerance of genotypes and separate the salt-tolerant genotypes from sensitive ones.

As the ranking of genotypes for salt tolerance can vary depending on the specific trait being considered, grouping genotypes based on multiple traits simultaneously is an efficient way to accurately determine the final ranking of the salt tolerance of genotypes. Therefore, employing suitable statistical methods is essential to achieve this objective. Heatmap clustering is one such method that provides a visual representation to rank the salt tolerance of genotypes based on multiple traits. This method facilitates grouping genotypes in a more straightforward manner, making the assessment of genotype performance under stress and non-stress conditions individually more practical and reliable. It also assists in grouping genotypes based on their performance levels under non-saline conditions (from high- to low-performing groups), while grouping genotypes based on their salt tolerance levels under salinity stress conditions (from salt-tolerant to salt-sensitive groups) [10,36]. Hence, heatmap clustering is a useful technique for determining the need for control treatment in evaluating the salt tolerance of genotypes using different agro-physiological traits. By classifying genotypes based on their performance under control and salinity stress conditions separately, and incorporating STIs that consider performance in both conditions, this method offers a thorough and precise evaluation of salinity tolerance.

Based on all of the foregoing information, we hypothesize that we can accurately assess the salt tolerance of wheat genotypes and classify the salt-tolerant genotypes from sensitive ones by analyzing agro-physiological traits data collected specifically under salinity stress conditions. It may not be necessary to include a control treatment when evaluating a large number of wheat genotypes. This hypothesis could not only lead to savings in effort, time, and resources when assessing a large number of genotypes but also improve our understanding of salt tolerance mechanisms based on physiological traits. Thus, the main objective of this research is to test this hypothesis. In order to accomplish this objective, the genotypes were grouped according to various agro-physiological traits assessed under normal and salinity stress conditions separately, as well as according to STIs of these traits, which consider the data from traits measured under both conditions.

## 2. Results

### 2.1. Analysis of Variance (ANOVA) of Different Agro-Physiological Traits

The combined analysis of variance (ANOVA) for two seasons revealed significant differences (*p* ≤ 0.05 and 0.001) in all agro-physiological traits across the seasons, salinity treatments, genotypes, and their potential interactions, with only a few exceptions (Table 1). ANOVA results indicated significant differences (*p* ≤ 0.05 and 0.001) among the main treatments (salinity and genotypes), except for K^+^ content, which did not show substantial differences between salinity treatments. The various agro-physiological traits also exhibited significant differences (*p* ≤ 0.05 and 0.01) in the interactions between the two main treatments (salinity treatments and genotypes), with the exception of the chlorophyll a/b ratio (Chla/Chlb) (Table 1). Statistically significant differences (*p* ≤ 0.05 and 0.01) were found between the seasons for all agro-morphological traits, except for Na^+^ and Ca^2+^ contents, green leaf area (GLA), and harvest index (HI). There was no significant impact on various yield traits, including grain number per spike (GNPS), grain yield (GY), biological yield (BY), and HI, when considering the interaction effects of season with salinity or genotype. Similarly, the interaction between seasons and salinity did not significantly affect Na^+^ content, Chla/Chlb ratio, total chlorophyll content (Chlt), and GLA. Additionally, the interaction between season and genotype did not significantly affect relative water content (RWC) and Chla/Chlb ratio. The three-way interaction (season, salinity, and genotype) did not significantly impact any growth and yield traits except for GLA, but did have a substantial effect on all ion and physiological traits except for RWC (Table 1).

### 2.2. Genotypic Performance Variation in Agro-Physiological Traits Under Control and Salinity Stress Conditions

Supplementary Figures S1–S3 display the genotypic performance for various agro-physiological traits under control and salinity stress conditions. Overall, most agro-physiological traits exhibited greater variability under salinity stress conditions compared to the control treatment. Salinity treatments led to a significant increase in Na^+^ content and CT, while causing a decrease in other agro-physiological traits compared to control treatments. Salinity stress resulted in a 1700% increase in Na^+^ content and a 35.0% increase in CT, as well as a significant decrease in K^+^/Na^+^ and Ca^2+^/Na^+^ ratios by approximately 95.0%. Physiological traits were reduced by 4–35%, while growth and yield traits decreased by 11–40% compared to the control treatment (Figures S1–S3). Furthermore, specific genotypes displayed minimal variation in the control treatment, but showed significant variation under salinity stress conditions (Figures S1–S3). Under normal conditions, the salt-sensitive genotype Sakha 61 showed comparable or superior performance to the salt-tolerant genotype Sakha 93 and moderately salt-tolerant genotype Sids1.

However, when exposed to salinity treatment, Sakha 61 exhibited lower performance for various agro-physiological traits than the other two genotypes. For instance, in comparison to Sakha 93 and Sids 1, Sakha 61 exhibited a 23.8% and 11.1% increase in Na^+^ content, a 6.6% and 6.5% increase in CT, and lower values ranging from 10.0% to 53.0% and 5.5% to 35.8% for other agro-physiological traits, respectively. Additionally, the Kawz genotype demonstrated superior performance for most traits under both conditions above compared to the three aforementioned genotypes (Figures S1–S3). Similarly, the different RILs displayed greater variability under both conditions, with specific lines showing better performance under control treatment and worse performance under salinity treatment. In contrast, the opposite was observed in other lines. Furthermore, some lines performed better under both treatments, while the opposite was true for other lines (Figures S1–S3).

As a result of these substantial differences in performance among genotypes under salinity and control conditions, the percentage change in traits due to salinity stress varied from one genotype to another (Table 2). Different genotypes exhibited varying degrees of reduction in response to salinity stress, with some experiencing a significant decrease, others a moderate decrease, and some only a slight decrease, as indicated by the color gradient in Table 2.

Regarding Na^+^ content and CT, different genotypes showed different levels of increase in both traits under salinity stress. The percentage of increase ranged from 1416.3% to 2484.0% for Na^+^ and from 23.9% to 49.0% for CT among genotypes. Specific genotypes exhibited an increase in K^+^ and Ca^2+^ levels, while others displayed a decrease in both traits when exposed to salinity stress. However, all genotypes had a greater reduction in K^+^/Na^+^ and Ca^2+^/Na^+^ ratios when exposed to salinity stress (Table 2).

### 2.3. Grouping Genotypes Based on Their Performance Under Control and Salinity Stress Individually, as Well as Stress Tolerance Indices

#### 2.3.1. Grouping Genotypes Based on Ionic Traits

**Figure 1:**
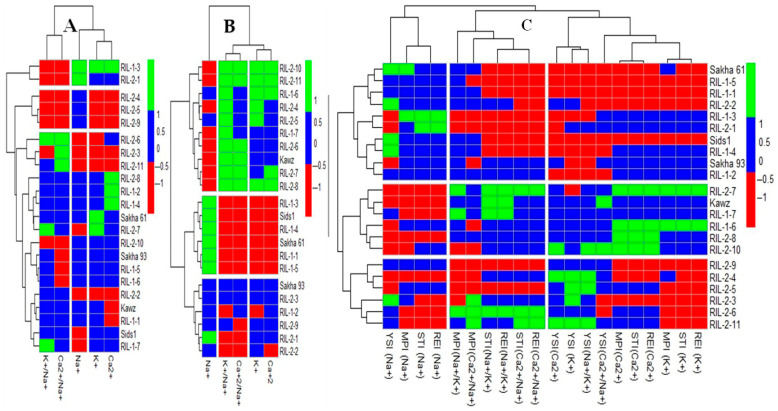
Grouping wheat genotypes based on the absolute values of ionic traits under control (**A**) and salinity stress (**B**) conditions separately, as well as based on different stress tolerance indices (STIs) of these traits (**C**). The full names of STIs are presented in Table 4 in the Materials and Methods section.

Figure 1A,B shows the grouping of cultivars/RILs based on their absolute values of ionic traits under control and salinity stress conditions. Figure 1C displays the grouping of cultivars/RILs according to their STIs for ionic traits, which shows the performance of genotypes for ionic traits under salinity stress relative to their performance under control conditions. When cultivars/RILs were grouped according to their ionic traits under salinity stress, they were divided into three groups, with the salt-tolerant Sakha 93 separated from the salt-sensitive Sakha 61 in two separate groups (Figure 1B). However, when cultivars/RILs were grouped based on their ionic traits under control or STIs, they were also split into three groups, but Sakha 93 and Sakha 61 were grouped in the same category (Figure 1A,C). When the Sakha 93 and Sakha 61 were separated based on ionic trait values under salinity stress, the group with Sakha 93 showed a 12.7% lower Na^+^ content and a 17.3% higher K^+^ content, a 7.9% higher Ca^2+^ content, a 26.9% higher K^+^/Na^+^ ratio, and an 18.5% higher Ca^2+^/Na^+^ ratio compared to the group with Sakha 61 (Figure 1B and Table 3). Additionally, the first group, consisting of Kawz, seven RILs from the RIL2 group, and two RILs from the RIL1 group, exhibited a 21.6% and 37.1% lower Na^+^ content and a 7.4–31.6% and 14.8–50.0% higher content for K^+^ and Ca^2+^, as well as their ratio with Na^+^, compared with the group with Sakha 93 and the group with Sakha 61, respectively (Figure 1B and Table 3). When Sakha 93 and Sakha 61 were grouped based on STIs of ionic traits, this group exhibited a 30.4% higher Na^+^ content and a 7.1–12.5% lower content for K^+^ and Ca^2+^, as well as their ratio with Na^+^, compared to the second group, which included the Kawz genotype (Figure 1C and Table 3). However, when all cultivars were grouped together based on ionic trait values under control conditions, this group exhibited a 34.1% decrease in Na^+^ content, an 8.1% decrease in K^+^ content, and a 4.2% decrease in Ca^2+^ content, but a 28.6% increase in K^+^/Na^+^ ratio and a 32.3% increase in Ca^2+^/Na^+^ ratio compared to the first group. Compared to the second group, this group showed a 10.3% decrease in Na^+^ content and an 8.1–35.3% increase in other ionic traits (Figure 1A and Table 3).

#### 2.3.2. Grouping Genotypes Based on Physiological Traits

**Figure 2:**
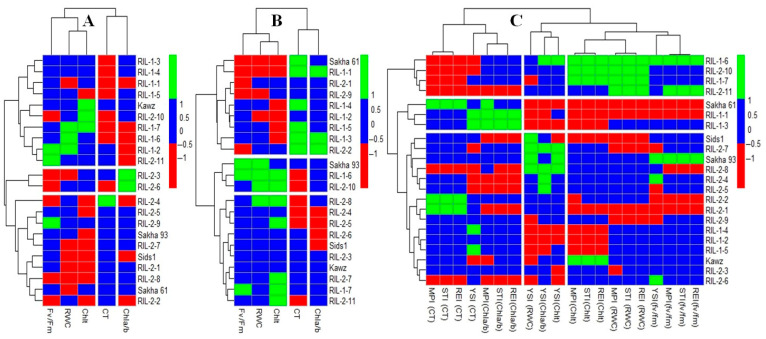
Grouping wheat genotypes based on the absolute values of physiological traits under control (**A**) and salinity stress (**B**) conditions separately, as well as based on different stress tolerance indices (STIs) of these traits (**C**). The full names of STIs are presented in Table 4 in the Materials and Methods section.

Heatmap clustering grouped genotypes into three distinct groups based on their physiological traits (RWC, CT, Fv/Fm, Chla/Chlb ratio, and Chlt) under control or salinity stress conditions (Figure 2A,B), as well as according to the STIs (Figure 2C). The heatmaps created with physiological trait values under salinity stress or STIs successfully differentiated between the salt-sensitive Sakha 61 and the salt-tolerant Sakha 93, while those generated using physiological trait values under control conditions failed to distinguish between the two genotypes. When cultivars/RILs were grouped based on physiological traits under control conditions (Figure 2A), the group with Sakha 93 and Sakha 61 exhibited comparable values for RWC, CT, and Fv/Fm and lower values for the Chla/Chlb ratio and Chlt compared to the other two groups (Table 3). When cultivars/RILs were grouped based on physiological traits under salinity stress conditions (Figure 2B), the group containing Sakha 93 exhibited a 7.2% increase in RWC, a 7.3% increase in Fv/Fm, a 29.5% increase in Chla/Chlb, and a 25.3% increase in Chlt, along with a 10.3% decrease in CT compared to the group containing Sakha 61 (Table 3).

While grouping cultivars/RILs based on STIs effectively distinguished Sakha 93 from Sakha 61 (Figure 2C), the differences between the two groups were not as significant as when both groups were separated based on physiological traits under salinity stress conditions. The group with Sakha 93 showed similar values for RWC, CT, and Fv/Fm, a 25.6% decrease in the Chla/Chlb ratio, and a 13.2% increase in Chlt compared to the group with Sakha 61 (Table 3). It is worth noting that the moderately salt-tolerant Sids 1 was clearly distinguished as a distinct group from Sakha 93 and Sakha 61 based on physiological traits under salinity stress. However, it was grouped with both genotypes when considering physiological traits under normal conditions, and with Sakha 93 when looking at STIs of physiological traits (Figure 2A–C). The group that contained Sakha 93 when cultivars/RILs were grouped based on physiological traits under salinity stress exhibited higher values for RWC, Fv/Fm, Chla/Chlb, and Chlt, and lower values for CT compared to the group containing Sakha 61 or the group containing Sids1. When the Sids 1 or Sakha 61 were grouped with Sakha 93 based on physiological traits under normal conditions or STIs, this group consistently showed similar or lower values for physiological traits compared to other groups (Figure 2C and Table 3).

#### 2.3.3. Grouping Genotypes Based on Growth and Yield Traits

**Figure 3:**
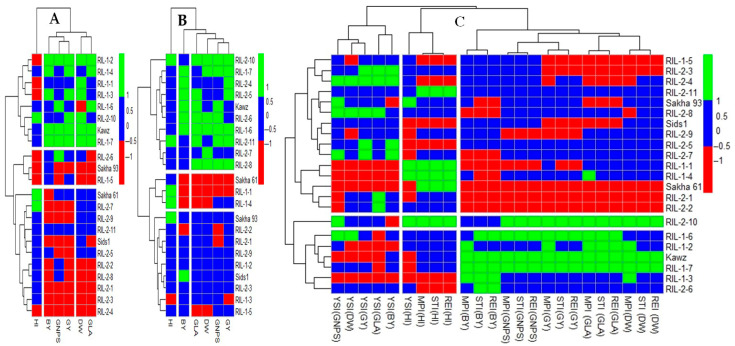
Grouping wheat genotypes based on the absolute values of growth, yield, and yield component traits under control (**A**) and salinity stress (**B**) conditions separately, as well as based on different stress tolerance indices (STIs) of these traits (**C**). The full names of STIs are presented in Table 4 in the Materials and Methods section.

Figure 3A,B displays the heatmap clustering of genotypes according to growth, yield, and yield component traits under both control and salinity stress conditions separately. In contrast, Figure 3C illustrates the heatmap clustering of genotypes based on STIs. The heatmaps generated using growth and yield trait values under control or salinity stress effectively distinguished between the salt-sensitive Sakha 61 and the salt-tolerant Sakha 93. However, the heatmap utilizing STIs of growth and yield traits did not clearly differentiate between the two genotypes. When cultivars/RILs were classified according to growth and yield traits under salinity stress conditions (Figure 3B), the group containing Sakha 93 displayed higher values for all growth and yield traits by 7.3–27.3%, except for HI, which had lower values by 23.4% compared to the group with Sakha 61 (Table 3). While grouping cultivars/RILs based on growth and yield traits under control conditions effectively distinguished Sakha 93 from Sakha 61 (Figure 3A), the group with Sakha 61 exhibited similar values for PDW, GLA, and GNPS, with a 5.1% decrease in GY, a 16.5% decrease in BY, and a 10.1% increase in HI compared to the group with Sakha 93 (Table 3). When Sakha 61 and Sakha 93 were grouped together according to STIs of growth and yield traits (Figure 2C), this group exhibited lower values for all growth and yield traits compared to the other two groups (Table 3).

The three heatmaps clearly showed that Kawz formed a distinct group separate from Sakha 93 and Sakha 61. This group exhibited significantly better performance in all growth and yield traits, with increases ranging from 5.1% to 33.0% and 8.0% to 31.3% compared to the Sakha 93 and Sakha 61 groups, respectively, when cultivars/RILs were grouped based on growth and yield traits under normal conditions (Figure 3A and Table 3). However, when grouped based on growth and yield traits under salinity stress conditions, this group only showed a 7.9% to 12.3% higher performance compared to the Sakha 93 group and a 15.3% to 33.0% higher performance compared to the Sakha 61 group (Figure 3B and Table 3). This group displayed significantly better performance in all growth and yield traits, with increases ranging from 7.8% to 18.7% compared to the group that included both Sakha 93 and Sakha 61 when cultivars/RILs were grouped based on STIs of growth and yield traits (Figure 3C and Table 3). It is worth noting that the Kawz group included six, two, and four RILs from the RIL1 group, and one, seven, and one RILs from the RIL2 group according to heatmap A (control conditions), B (salinity stress conditions), and C (STIs for traits), respectively.

## 3. Discussion

Plant breeders frequently take into account various physiological traits, including the concentrations of toxic ions (Na^+^) and their balance with essential ions (K^+^ and Ca^2+^), RWC, Chlt, and chlorophyll fluorescence, when assessing the salt tolerance of genotypes and selecting genetic materials for breeding initiatives aimed at enhancing salt tolerance. This is because these traits provide a comprehensive understanding of how genotypes can withstand salinity stress [36,37,38,39]. However, evaluating salt tolerance for many genotypes using these traits can be expensive, laborious, and time-consuming, and requires expertise and careful attention. Moreover, it is difficult to ignore the importance of agronomic traits, such as plant dry matter measured at different growth stages and grain yield and its components, as key screening criteria for evaluating the salt tolerance of wheat genotypes. This is because these traits are typically the primary consideration when assessing the salt tolerance of genotypes in actual field conditions, as they directly impact agricultural productivity [39,40]. Although measuring different agronomic traits may not require specialized expertise and meticulous attention, evaluating salt tolerance for a large number of genotypes using these traits can be expensive, laborious, and time-consuming. Hence, the hypothesis of our study is that we can assess the salt tolerance of wheat genotypes by analyzing agro-physiological traits solely under salinity stress conditions, thereby eliminating the necessity to measure these traits under control conditions. If validated, this approach could reduce the time and expense needed for evaluating salt tolerance by 50% without compromising precision. To test this hypothesis, 22 wheat genotypes with varying levels of salt tolerance were selected as plant materials and evaluated under standard and high salinity conditions in real field settings. The ANOVA results of this study showed that the salinity treatment significantly impacted the agro-physiological traits studied. Additionally, there was a notable genotypic effect on most agro-physiological traits (Table 1). These results indicate that the agro-physiological traits analyzed in this study can be used as effective screening criteria to evaluate the salt tolerance of wheat genotypes under real field conditions. However, is it necessary to evaluate these traits only under salinity stress conditions to accurately assess the salt tolerance of genotypes and reduce the time and expense required for evaluating salt tolerance by 50%, or should we also assess these traits under control conditions? This will be discussed in the following sections.

Previous studies have shown that various ionic characteristics, such as levels of toxic ions (Na^+^) and essential ions (K^+^ and Ca^2+^), along with the ratio between these ions, can be valuable indicators for assessing salt tolerance in genotypes and distinguishing between salt-tolerant and salt-sensitive genotypes. Because Na+ toxicity is the primary physiological constraint affecting crop performance under prolonged exposure to salinity, the salt tolerance of salt-tolerant genotypes is usually linked to their ability to reduce Na^+^ accumulation in plant tissues. Therefore, studies have indicated that salt-tolerant genotypes typically show lower Na^+^ levels but higher levels of K^+^ and Ca^2+^, along with increased K^+^/Na^+^ and Ca^2+^/Na^+^ ratios under salinity stress conditions [12,13,14,15,36,40]. Under non-saline conditions, Na^+^, K^+^, and Ca^2+^ levels and proportions in the shoots remain relatively stable across different wheat genotypes. However, when exposed to saline stress, there are significant changes in the levels and proportions of these elements among genotypes (Figure S1). This suggests that the ion content values obtained only under salinity stress conditions may be enough to effectively evaluate the salt tolerance of genotypes. In this study, when grouping genotypes based on their ionic contents under salinity stress conditions, the salt-tolerant Sakha 93 was clearly distinguished from the salt-sensitive Sakha 61. Additionally, the group with Sakha 93 exhibited lower levels of Na^+^ and higher levels of K^+^ and Ca^2+^, along with higher K^+^/Na^+^ and Ca^2+^/Na^+^ ratios compared to the group with Sakha 61 (Figure 1B and Table 3). However, when genotypes are categorized according to ionic traits under normal conditions or STIs, they fail to differentiate between Sakha 93 and Sakha 61 as distinct groups. Instead, both genotypes are clustered together, and this merged group exhibits higher or intermediate levels of ionic traits compared to the other two groups (Figure 1A,C and Table 3). These findings confirm that assessing ion contents under salinity stress alone can accurately evaluate the salt tolerance of genotypes, eliminating the necessity to measure ion contents under control conditions. This approach offers benefits such as a 50% reduction in ion estimation costs and enhanced understanding of the salt tolerance mechanisms associated with ionic characteristics. An analysis of ionic characteristics in the Sakha 93 group and the Sakha 61 group (Table 3) reveals that salt tolerance mechanisms in wheat usually exclude Na^+^ to avoid excessive buildup in the shoot, as well as to maintain appropriate K^+^/Na^+^ and Ca^2+^/Na^+^ ratios. Previous studies on wheat and other cereal crops have also shown similar results, indicating that salt tolerance in these crops is associated with Na^+^ exclusion, which leads to lower shoot Na^+^ contents and improved K^+^/Na^+^ and Ca^2+^/Na^+^ ratios [13,14,16,23,24,31,37]. Xue et al. [41] also found that transgenic wheat lines containing NHX1 from *Arabidopsis thaliana* showed a 65% decrease in leaf Na^+^ levels and a 176% increase in leaf K^+^ levels compared to wild-type plants.

When genotypes were grouped based on their ionic traits under salinity stress conditions, it was noted that some RILs in the Sakha 93 group displayed higher Na^+^ levels and lower K^+^/Na^+^ and Ca^2+^/Na^+^ ratios, similar to those in the Sakha 61 group (Figure S1). This indicates that salt tolerance in wheat genotypes is not solely dependent on their ability to exclude Na^+^ from shoots. Instead, the salt tolerance in wheat genotypes is also associated with tissue tolerance, which is achieved through the sequestration of Na^+^ in vacuoles and using it as an economical osmotic adjustment. This mechanism helps to prevent the accumulation of Na^+^ to harmful levels in actively metabolizing tissues [36,40,41]. This means that various combinations of Na^+^ exclusion and tissue tolerance mechanisms can result in similar levels of salt tolerance in wheat genotypes. Therefore, classifying genotypes according to their ionic characteristics under salinity stress, specifically Na^+^ and K^+^ levels, not only distinguishes salt-tolerant from salt-sensitive genotypes but also provides insights into the mechanisms of salt tolerance in wheat genotypes.

Many physiological traits are interrelated, particularly under stressful conditions, where alterations in one trait frequently lead to notable changes in others. As a result, these interlinked traits are commonly utilized as effective screening criteria for evaluating the stress tolerance of various genotypes. For instance, the rapid closure of stomata due to the osmotic and ionic effects of salinity stress results in decreased transpiration rates necessary for canopy cooling, leading to a notable increase in CT [18,19]. Stomatal closure is a protective mechanism that helps minimize water loss in response to the negative osmotic effects of salinity stress, thereby maintaining optimal cell hydration and preserving RWC. Stomatal closure not only decreases photosynthesis rates by altering the CO_2_/O_2_ ratio in leaves but also affects the bio-energetic processes of photosynthesis, resulting in an excess production of reactive oxygen species (ROS) [42,43]. This increased ROS production can damage the structure and function of chloroplasts, ultimately leading to a decrease in overall chlorophyll content (Chlt) and chlorophyll fluorescence [16,20,21,44]. Most notably, many physiological traits exhibit strong correlations with each other under salinity stress conditions, whereas this correlation tends to weaken or become insignificant under normal control conditions [9,16]. The strong correlation and interaction among physiological traits under salinity stress conditions validate the effectiveness of these traits as screening criteria for accurately evaluating the salt tolerance of wheat genotypes. In this study, while grouping genotypes based on physiological traits (RWC, CT, Chlt, Chla/Chlab, and Fv/Fm) under control, salinity stress, or STIs successfully differentiated between the salt-tolerant Sakha 93 genotype and the salt-sensitive Sakha 61 genotype (Figure 2A–C), the three heatmap clusters varied significantly in their ability to assess the salt tolerance of genotypes (Table 3). When classifying genotypes based on their physiological traits under control or STIs, the Sakha 61 group exhibited similar or higher values for the tested physiological traits compared to the Sakha 93 group. However, under salinity stress conditions, the Sakha 93 group had a lower value for CT and higher values for other traits compared to the Sakha 61 group (Table 3). These findings demonstrate that grouping genotypes based on physiological traits under salinity stress alone is effective not only for accurately assessing salt tolerance in wheat genotypes but also for understanding the mechanisms of salt tolerance through multiple physiological traits. Therefore, measuring these traits under salinity conditions may be sufficient without the need to measure them under control conditions. These results could be attributed to the fact that the genotypes grown under salinity stress conditions showed notable differences in their physiological traits (Figure S2), likely due to the susceptibility of physiological characteristics to the osmotic and ionic impacts of salinity stress. However, the range of physiological traits among genotypes in non-saline conditions is typically minimal or insignificant (Figure S2). Therefore, the significant differences in physiological characteristics among genotypes under salinity stress enable a more precise and effective evaluation of salt tolerance based on these traits. For instance, previous studies have indicated that the RWC exhibited significant variability among genotypes when exposed to salinity stress, with genotypes showing higher salt tolerance also displaying higher RWC levels. However, differences in RWC among genotypes under normal conditions were not found to be significant [23,24,45,46,47,48,49,50,51]. Salt-tolerant genotypes can maintain a high RWC under salinity stress by accumulating organic and inorganic compounds to facilitate osmotic adjustment. On the other hand, salt-sensitive genotypes may face challenges in achieving osmotic adjustment under salinity stress [52]. Thus, assessing the RWC of genotypes under salinity stress is valuable for evaluating their salt tolerance accurately and understanding the mechanisms of salt tolerance through water balance under salinity stress.

In high salinity stress conditions, various reasons contribute to a significant reduction in Chlt and Fv/Fm, along with an elevation in plant CT in genotypes, which are not typically observed in non-saline conditions. For instance, the accumulation of Na^+^ and ROS in leaf tissues is a common occurrence under a high salinity stress level, especially in salt-sensitive genotypes. This often leads to alterations in chlorophyll pigments, an increased activity of chlorophyll-degrading enzymes, and/or a restriction in chlorophyll production, ultimately resulting in leaf chlorosis [53,54]. Moreover, an excess of Na^+^ in leaf tissues can lead to stomatal closure, which can disturb the equilibrium between light absorption in photosystem II (PS II) and energy utilization [44,45]. In non-saline conditions without osmotic stress, stomata open to facilitate evaporative cooling and reduce CT. However, under the osmotic effects of salinity stress, stomata quickly close to avoid excessive water loss, even at the expense of evaporative cooling, leading to higher CT [18]. The different reasons mentioned above indicate that assessing Chlt, Fv/Fm, and CT under salinity stress conditions could be enough to assess the salt tolerance of genotypes, eliminating the need to evaluate them under normal conditions. This is reinforced by the findings shown in Figure 2A–C and Table 3.

Assessing the salt tolerance of genotypes also requires examining their growth and yield characteristics, which reveal the relationship between salinity stress and the resulting physiological and biochemical changes at the plant level. Additionally, growth traits are prioritized as primary goals, especially in stressful conditions, as they have a significant impact on final GY [39]. Several studies have shown that traits related to plant growth, such as GLA and PDW, are essential for assessing the salt tolerance of various genotypes, especially when assessed at different growth stages [10,12,52]. The inhibition of leaf growth is a common initial response to salt stress, especially in salt-sensitive genotypes exposed to high salinity levels [55]. Saqib et al. [56] found that spikelets per spike and grain weight are the key yield components in wheat that are highly responsive and sensitive in determining salt tolerance in wheat genotypes. The detrimental negative impacts of ionic and osmotic aspects of salinity stress, including diminished leaf development or longevity, reduced photosynthetic ability, lower fertilization rates, and hindered transport of photo-assimilates from source to sink, result in a substantial reduction in final GY [11,16,55]. Based on the information provided, growth and yield traits can serve as effective screening criteria for assessing the salt tolerance of genotypes. Previous studies have utilized the reduction percentage of these traits under salinity stress compared to control treatments or calculated the STIs of these traits when evaluating the salt tolerance of genotypes. However, both methods necessitate measuring the traits under both control and salinity stress conditions. If the performance of genotypes varies significantly between control and salinity stress conditions as shown in Table 2 and Figure S3, neither method may provide an accurate assessment of salt tolerance. In this study, grouping genotypes based on growth and yield characteristics under different conditions (control, salinity stress, or STIs) effectively differentiated between the salt-tolerant Sakha 93 genotype and the salt-sensitive Sakha 61 genotype (Figure 2A–C). Notably, the Sakha 93 group consistently exhibited superior growth and yield traits compared to the Sakha 61 group when genotypes were classified based on these traits under salinity stress. Conversely, when genotypes were classified based on these traits under control conditions or STIs, the Sakha 61 group demonstrated comparable or higher values for growth and yield components than the Sakha 93 group (Table 3). These findings indicate that assessing growth and yield components under salinity stress conditions alone may be enough to evaluate the salt tolerance of genotypes, without the need to evaluate them under normal conditions. This finding was probably due to certain genotypes having characteristics that allow them to withstand and adjust to salt stress, resulting in successful growth and productivity in saline environments. Nevertheless, these characteristics may not always result in optimal growth and productivity for the same genotypes under normal conditions. For instance, the ability of certain genotypes to maintain open stomata for increased carbon dioxide absorption, coupled with a large leaf area for enhanced photosynthetic efficiency, is a key trait that typically results in improved growth and productivity in optimal conditions. However, in the presence of salt stress, these characteristics can have a detrimental effect on growth and productivity due to the osmotic pressure of salinity, which impedes water uptake. On the other hand, osmotic adjustment through the accumulation of compatible solutes in the cytosol is a strategy employed by plants to enhance growth and productivity under high salt stress conditions. Nevertheless, this approach may not be practical in non-saline conditions because of the significant energy required for osmotic adjustment, which could hinder growth and overall productivity. Therefore, genotypes that are specifically bred for optimal growth under non-stressful conditions may lack important salt-tolerance mechanisms, such as efficient Na^+^ exclusion and osmotic adjustment, due to the trade-off between growth and stress adaptation. As a result, these high-yielding genotypes often exhibit poor performance under salinity stress compared to salt-tolerant genotypes that possess these protective traits. Conversely, salt-adapted genotypes may have lower yield potential under ideal conditions, as they must allocate resources to maintain stress-responsive pathways [39]. Thus, the traits that contribute to a genotype’s performance under optimal conditions differ from those that confer tolerance to salt stress. These findings confirm that evaluating agro-physiological traits under salinity stress conditions may be sufficient to assess the salt tolerance of genotypes, eliminating the need for measurements under normal conditions.

## 4. Materials and Methods

### 4.1. Experimental Setup Details

**Figure 4:**
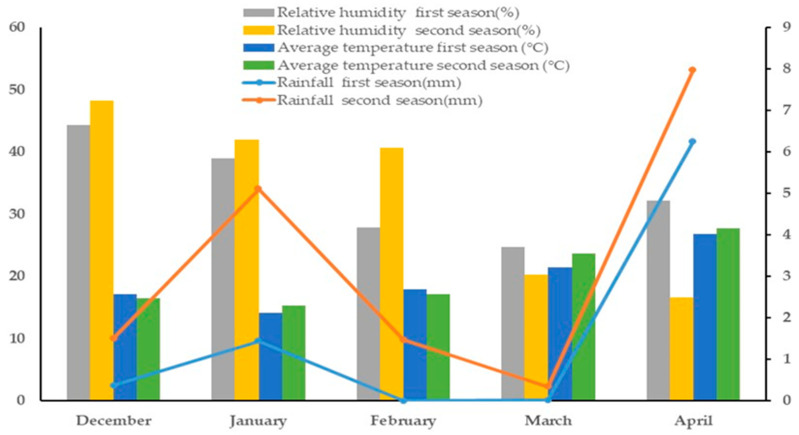
Climatic data collected by an automatic weather station located near the experimental station throughout the wheat growing season in both the first and second seasons.

The study’s experiments took place in actual field conditions over two consecutive seasons, 2019/2020 and 2020/2021, at the Dierab experimental station of the Plant Production section within the College of Food and Agricultural Sciences at King Saud University in Riyadh Governorate, Saudi Arabia (24°25′ N, 46°34′ E). The specific climatic conditions at the experimental station during the wheat growing season are illustrated in Figure 4.

The soil has a sandy loam texture with 65.5% sand, 24.1% silt, and 10.4% clay. It also contains 0.46% organic matter, has a bulk density of 1.48 g cm^−3^, a pH of 7.85, an electrical conductivity (EC) of 1.12 dS m^−1^, and an available N, P_2_O_5_, and K_2_O of 45.2, 2.44, and 186.9 mg kg^−1^, respectively.

The study evaluated the genotypes of three parents (Sakha 61, Sakha 93, and Sids 1) and their 18 F_8_ recombinant inbred lines (RILs), along with a commercial genotype Kawz. The RILs included 7 lines from a cross between the salt-sensitive genotype Sakha 61 and the salt-tolerant genotype Sakha 93 (RIL1-group), and 11 lines from a cross between Sakha 93 and the moderately salt-tolerant genotype Sids 1 (RIL2-group). The salt tolerance of the three parents had been previously evaluated in various experiments conducted under greenhouse and real field conditions using different agro-physiological traits [28,37,57].

**Figure 5:**
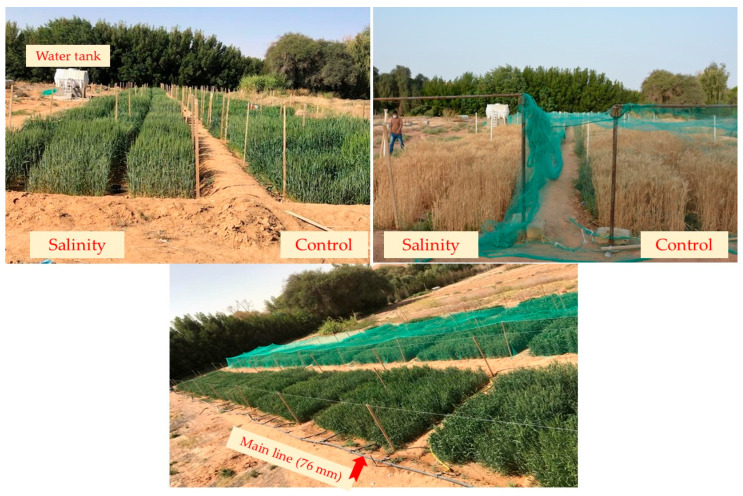
Plot layouts of genotypes grown under control and salinity conditions at different growth stages of wheat.

The genotypes were evaluated in a randomized block design with a split-plot arrangement and three replications, under non-saline (control) and high saline level (150 mM NaCl) conditions. The salinity treatments were allocated to the main plot, and the genotypes were randomly distributed among the subplots. All genotypes were first irrigated with non-saline water for 21 days to establish the seedlings. Afterward, the genotypes in the control group continued to be irrigated with non-saline water, while those in the salinity treatment group were irrigated with saline water containing a solution of 150 mM NaCl L^−1^. The low-pressure surface irrigation system was used to apply irrigation water. In the salinity treatment, this system included a main line with a 76 mm diameter. To control the amount of saline water applied to each subplot, this main line was connected to 5.0 m^3^ water tank, branched off to sub-main hoses, and equipped with a manual control valve at each subplot. The irrigation system for the control treatment is identical to the salinity treatment, except that the main line connects directly to the water irrigation source (Figure 5). Irrigation frequency and rate for both control and irrigation treatment were determined based on meteorological data from the experimental station and the growth stages of the wheat crop. According to this data, approximately 0.5 m^3^ m^−2^ of water was applied. This amount of water was applied 10 times throughout the entire growth period of the wheat genotypes.

Seeds of each genotype were manually sown in five rows, each 1.5 m long and spaced 20 cm apart at a rate of 150 kg ha^−1^ on 25 November in the first year and 17 November in the second year. All genotypes were fertilized with 150 kg ha^−1^ of nitrogen (N), 100 kg ha^−1^ of phosphorus (P), and 90 kg ha^−1^ of potassium (K). The NPK fertilizers used were ammonium nitrate (33.5% N), calcium superphosphate (18.5% P_2_O_5_), and potassium chloride (50% K_2_O). The entire amount of P and half of the K were applied at sowing, while the remaining half of K was applied at the late booting growth stage. The N fertilizer was split into three equal doses, with one dose applied at sowing, one at tillering, and one at the late booting growth stage.

### 4.2. Agro-Physiological Traits Measurements

#### 4.2.1. Determination of Ion and Physiological Traits

To determine the concentrations of ion content (Na^+^, K^+^, and Ca^2+^), 0.4 g of finely ground plant samples collected at the harvest stage were placed in digestion tubes containing a mixture of 2 mL of perchloric acid (HClO_4_) and 8 mL of concentrated nitric acid (HNO_3_) and left overnight. The mixture was heated at 300 °C for 4 h, then allowed to cool to room temperature before being transferred to a 50 mL volumetric flask and adjusted to a final volume of 50 mL with distilled water. A 10 mL sample was used to measure the concentrations of the three elements using a flame photometer (ELEX 6361, Eppendorf AG, Hamburg, Germany) and subsequently, the K^+^/Na^+^ and Na^+^/Ca^2+^ ratios were calculated.

The physiological traits, including relative water content (RWC), chlorophyll content, maximal PSII efficiency (Fv/Fm), and canopy temperature (CT), were measured at 75 days after sowing, specifically during the flowering growth stage. The topmost fully expanded leaves were randomly selected to measure the first three physiological traits. Leaf samples of 20 cm^2^ were cut from these leaves, and their fresh weight (FW) was immediately measured. The samples were immersed in distilled water for 24 h at 25 °C in dark conditions; then, excess water was removed from the leaf surface, and their turgid weight (TW) was recorded. Subsequently, the leaf samples were dried in an oven at 75 °C for 72 h and weighed to determine their dry weight (DW). The weights obtained were used in the following equation to calculate the RWC:

RWC = (FW − DW)/(TW − DW) × 100(1)

Leaf samples of 0.4 g were cut from the topmost fully expanded leaves and soaked in 5 mL 80% acetone (*v*/*v*) in dark conditions until the leaf samples were fully bleached. The extracted solutions were centrifuged at 400 rpm for 5 min and then transferred to a tube to be diluted to a final volume of 15 mL with 80% acetone. The absorbance of the solutions was measured at 645 nm (A645) and 663 nm (A663) using a spectrophotometer (UV-2550, Shimadzu, Tokyo, Japan). Finally, the concentrations of different photosynthetic pigments, in mg per g fresh weight (mg g^−1^ FW^−1^), were calculated using the equations provided by Lichtenthaler and Wellburn [58]:

Chla = (12.72 × A663) − (2.69 × A645) × V/FW(2)

Chlb = (22.87 × A645) − (4.67 × A663) × V/FW(3)

Chlt = [(8.02 × A663) + (20.21 × A645)] × V/FW(4)

where V is the final volume of the extract solution and FW is the fresh weight of leaf samples.

The Fv/Fm ratio was measured on the topmost fully expanded leaves using a portable chlorophyll fluorometer (Photon Systems Instruments, PAM-2100, Walz, Germany). The minimum fluorescence (F0) was measured in selected dark-adapted leaves for 30 min using light exclusion clips and measured with low-intensity modulated light (<0.1 mmol m^−2^ s^−1^) to ensure consistent fluorescence readings. The maximum fluorescence (Fm) was recorded using a saturating light intensity of 8000 mmol m^−2^ s^−1^ for 0.8 s on a leaf that had been dark-adapted. The Fv/Fm ratio in the dark-adapted state was calculated using the following formula:

Fv/Fm = (Fm − F0)/Fm(5)

The CT was measured between 10:00 and 13:00 under clear-sky conditions using a handheld infrared thermal camera (Therma CAM SC 3000 infrared camera, FLIR System, Wilsonville, OR, USA). The camera had a resolution of 640 × 480 pixels and a wide-angle lens field of view of 45° × 34°. It had a thermal sensitivity of ≤0.05 °C at 30 °C and an accuracy of ±0.2 °C within the temperature range of −20 °C to 600 °C. It operated in a wavelength range of 7.5–14 µm with a focal length of 13 mm. Emissivity was set to 0.95 for wet leaves and 0.96 for dry leaves [59]. To account for fluctuations in ambient temperature and other sources of non-uniform noise, the camera underwent calibration using a temperature-dependent radiometric matrix before taking measurements. To effectively capture thermal images of the canopy, the camera was positioned vertically about 80 cm above the canopy in a nadir orientation. The FLIR Research Pro Software version 5.1 (FLIR System, Wilsonville, OR, USA) was used to calculate the average leaf temperature in each thermal image by averaging the temperature within a polygon area fitted around the selected canopy area.

#### 4.2.2. Determination of Agronomic Traits

Green leaf area (GLA) and plant dry weight (PDW) were assessed 75 days after sowing, specifically during the flowering growth stage. Ten plants from each genotype were randomly selected, and their green leaves were separated and measured for leaf area using a leaf area meter (LI 3100; LI-COR Inc., Lincoln, NE, USA). Subsequently, all parts of the ten plants (leaves, stems, and spikes) were dried collectively at 75 °C for 72 h until a constant weight was reached.

At the maturity growth stage, which occurred on 24 April in the first season and 21 April in the second season, 20 spikes from each genotype were randomly selected. The grains were subsequently separated and weighed to calculate the grain number per spike (GNPS). Finally, the plants from the central three rows, each 1.25 m long (0.75 m^2^ total area), of each genotype, were hand-harvested, air-dried for 7 days, and weighed to determine their biological yield (BY). The grains were then threshed, cleaned, and adjusted to a moisture content of 14.0% before being weighed and recorded as grain yield (GY). The harvest index (HI) was calculated by dividing GY by BY.

### 4.3. Determination of Stress Tolerance Indices

The stress tolerance indices (STIs) shown in Table 4 were calculated to evaluate the genotypes’ performance under salinity stress relative to non-stress conditions.

### 4.4. Data Analysis

To examine the effects of year, salinity, genotypes, and their possible interactions, the data for each agro-physiological trait were subjected to an analysis of variance (ANOVA) suitable for a split-plot design in a randomized complete block design using the statistical software package COSTAT 6.451 for Windows (Cohort software, Berkeley, CA, USA). Before the analysis, the data for all agro-physiological traits were initially examined for normality to detect and eliminate any outliers. Subsequently, a Bartlett test was performed to evaluate the homogeneity of variance for each trait to determine if the data from both seasons could be combined for analysis. Multiple heatmaps were created to categorize genotypes according to their ion contents, physiological traits, and agronomic traits under normal and salinity stress conditions. Additionally, genotypes were grouped based on stress tolerance indices (STIs), which consider their performance under stress compared to non-stress conditions. The heatmaps were created using R statistical software (RStudio version 4.2.2, Boston, MA, USA) using data combined from both years. The pheatmap package (version 1.0.12) was utilized for generating the primary heatmap visualization.

## 5. Conclusions

The findings of this study suggest that evaluating the salt tolerance of wheat genotypes in actual field conditions can be achieved by focusing on agro-physiological traits, especially under salinity stress, particularly ionic and physiological traits, which are both labor-intensive and costly to assess. This approach could eliminate the need to test genotypes under control conditions, which can be time-consuming and expensive when dealing with a large number of genotypes. Furthermore, assessing genotypes under salinity stress alone also saves time, resources, and money and provides valuable insights into the mechanisms of salt tolerance in wheat genotypes. Grouping genotypes based on absolute values of various agro-physiological traits under salinity stress conditions consistently and effectively distinguished between the salt-tolerant Sakha 93 genotype and the salt-sensitive Sakha 61 genotype as well as explained the mechanism of salt tolerance of wheat genotypes. However, grouping them based on absolute values under control conditions or STIs sometimes fails to differentiate between the two genotypes and does not help us to understand the mechanisms of salt tolerance. When grouping genotypes according to their responses to salinity stress, the group that included Sakha 93 consistently showed lower Na^+^ content and CT, but higher values for other agro-physiological traits. In contrast, the group containing Sakha 61 exhibited the opposite pattern. However, when grouping genotypes based on their responses to control conditions or STIs, the group with Sakha 61 displayed similar or higher values for most agro-physiological traits compared to Sakha 93. Most importantly, the mechanisms of salt tolerance in genotypes are well understood based on their ionic and physiological traits when grouped according to their responses to salinity stress. In conclusion, assessing ionic and agro-physiological traits under salinity stress alone can accurately assess the salt tolerance of wheat genotypes in real field conditions and offer valuable insights into their salt tolerance mechanisms, eliminating the necessity for evaluations under control conditions. This approach can simplify the process and conserve time, effort, and resources when assessing the salt tolerance of various genotypes in a breeding program.

## Figures and Tables

**Table 1:** Mean square values of combined analysis to show the impact of season (S), salinity treatments (STs), genotypic effect, and their interactions on individual agro-physiological traits.

Traits	Source	S	ST	S × ST	G	S × G	ST × G	S × ST × G	Error
	DF	1	1	1	21	21	21	21	168
Ionic traits	Na^+^	57,187.8 ^ns^	192,392,393.3 ***	72,959.8 ^ns^	232,724.7 ***	58,695.9 ***	205,223.5 ***	56,381.8 ***	24,694.8
	K^+^	2,723,428.9 ***	4725.2 ^ns^	1,463,895.5 ***	102,353.4 ***	23,520.6 ***	298,397.5 ***	32,574.6 ***	7188.9
	Ca^2+^	11.5 ^ns^	295,099.3 ***	66,240.7 ***	6651.3 ***	2989.8 ***	7116.7 ***	6613.0 ***	440.8
	K^+^/Na^+^	22.24 ***	6782.14 ***	9.31 ***	13.01 ***	0.61 ***	13.30 ***	0.46 ***	0.18
	Ca^2+^/Na^+^	0.47 ***	1332.72 ***	0.61 ***	1.91 ***	0.20 ***	1.68 ***	0.22 ***	0.07
Physiological traits	RWC	55.47 *	13,571.52 ***	352.16 ***	27.18 ***	10.40 ^ns^	22.84 ***	6.71 ^ns^	10.20
	CT	298.05 ***	5842.01 ***	48.12 ***	9.92 ***	1.95 ***	10.17 ***	1.72 ***	0.49
	Fv/Fm	0.0582 ***	0.0740 ***	0.0032 *	0.0033 ***	0.0031 ***	0.0031 ***	0.0016 ***	0.0006
	Chla/Chlb	2.50 ***	136.28 ***	4.50 ^ns^	0.21 ^ns^	0.18 ^ns^	0.24 ^ns^	0.25 ^ns^	0.16
	Chlt	7.44 ***	25.90 ***	4.52 ^ns^	1.80 ***	0.33 ***	1.61 ***	0.25 ***	0.06
Growth and yield traits	PDW	2.92 ***	159.82 ***	2.55 *	2.14 ***	0.43 ***	2.17 ***	0.24 ^ns^	0.18
	GLA	461.3 ^ns^	568,570.8 ***	258.6 ^ns^	10,254.4 ***	2146.4 ***	9181.7 ***	2254.8 ***	424.4
	GNPS	288.94 ***	9018.02 ***	23.26 ^ns^	64.32 ***	8.22 ^ns^	24.12 ***	4.13 ^ns^	7.75
	GY	52,714.8 ***	2,463,835.2 ***	1367.8 ^ns^	17,035.4 ***	2114.0 ^ns^	13,600.9 ***	825.0 ^ns^	1440.4
	BY	789,014.9 ***	11,952,099.4 ***	15,173.6 ^ns^	244,345.6 ***	10,232.4 ^ns^	265,009.3 ***	14,992.5 ^ns^	22,409.3
	HI	11.52 ^ns^	902.69 ***	24.24 ^ns^	65.49 ***	11.52 ^ns^	45.98 ***	9.89 ^ns^	12.52

The abbreviations of Na^+^, K^+^, Ca^2+^, RWC, CT, Fv/Fm, Chla/Chlb, Chlt, PDW, GLA, GNPS, GY, BY, and HI indicate content of sodium, potassium, and calcium (mmol kg^−1^ DW), relative water content (%), canopy temperature (°C), maximum quantum PSII photochemical efficiency, chlorophyll a/b ratio, total chlorophyll content (mg g^−1^ FW), plant dry weight (g plant^−1^), green leaf area (cm^2^ plant^−1^), grain number per spike, biological yield (kg m^2^), grain yield (kg m^2^), and harvest index (%), respectively. * *p* ≤ 0.05, *** *p* ≤ 0.001, and ns—not significant.

**Table 2:** Decrease or increase in the percentage of various agro-physiological traits under high salinity levels compared to the control treatment for each genotype.

Genotypes	Na^+^	K^+^	Ca^2+^	K^+^/Na^+^	Ca^2+^/Na^+^	RWC	CT	Fv/Fm	Chla/Chlb	Chlt	PDW	GLA	GNPS	GY	BY	HI
Sakha 93	−1587.0	16.7	−16.3	95.1	93.1	13.5	−31.8	−1.7	−31.0	22.0	18.6	31.1	17.5	27.7	33.3	−8.5
Sids1	−2208.5	12.5	−3.3	96.2	95.5	12.7	−31.6	3.2	10.1	31.9	20.1	28.9	22.1	30.8	11.4	21.2
Sakha 61	−2276.8	39.8	1.6	97.5	95.8	21.5	−39.5	11.2	72.3	40.7	41.4	51.3	32.6	47.3	33.5	20.8
Kawz	−1488.0	−1.7	−25.4	93.7	92.1	19.8	−28.9	3.7	17.1	31.4	34.5	48.8	26.7	40.0	26.0	19.1
RIL1-1	−2161.2	28.2	−6.2	96.8	95.3	21.3	−49.0	9.4	52.3	27.5	47.0	49.3	32.4	44.8	51.7	−15.4
RIL1-2	−1822.9	29.4	−6.9	96.3	94.3	23.3	−37.8	5.9	50.5	32.8	35.2	59.5	24.4	45.6	38.8	11.2
RIL1-3	−1454.1	32.0	1.6	95.6	93.7	20.1	−45.4	4.2	57.1	31.6	41.2	52.5	29.5	45.7	33.2	18.0
RIL1-4	−2223.4	23.8	−1.7	96.7	95.6	19.8	−47.4	3.0	56.4	38.9	43.4	60.0	29.6	45.5	52.6	−15.9
RIL1-5	−1877.8	36.1	2.0	96.8	95.0	19.5	−44.3	5.9	35.4	30.1	32.9	36.1	21.9	34.9	27.0	10.6
RIL1-6	−474.1	−2.2	−21.6	93.5	92.3	17.0	−28.4	−1.8	−42.4	17.3	11.4	48.2	17.2	30.0	22.7	9.2
RIL1-7	−1783.6	−0.7	−17.1	94.3	93.5	20.5	−38.8	0.6	4.3	24.1	31.1	52.3	21.2	37.5	21.4	20.7
RIL2-1	−1468.4	2.1	−10.1	93.7	93.0	17.5	−33.7	8.6	−1.3	27.9	20.4	16.0	26.0	30.4	11.9	20.1
RIL2-2	−2279.2	−3.3	−16.2	95.7	95.1	17.2	−37.0	9.3	18.4	23.7	20.9	14.2	28.6	32.3	24.1	9.6
RIL2-3	−2484.0	−67.3	−27.4	93.6	95.0	16.2	−38.1	1.3	3.4	32.1	13.7	5.9	20.0	23.0	4.6	17.6
RIL2-4	−1671.6	−63.0	−33.9	90.8	92.4	17.8	−23.9	−0.6	−57.5	24.8	8.2	11.7	17.5	17.3	−1.2	17.7
RIL2-5	−1512.9	−68.5	−24.9	89.6	92.2	15.6	−27.0	6.2	−54.7	27.1	22.5	37.4	21.5	23.4	6.2	18.5
RIL2-6	−2055.1	−13.1	−16.8	94.5	94.5	19.8	−38.0	−0.2	11.7	38.2	17.4	23.2	23.3	33.7	19.8	17.5
RIL2-7	−1598.6	9.6	−18.2	94.6	93.0	14.0	−28.3	6.4	−54.4	20.1	18.2	42.8	16.1	23.6	7.1	18.4
RIL2-8	−1468.9	−13.2	−12.3	92.8	92.8	11.0	−26.0	2.1	−60.5	21.7	6.4	18.8	15.9	21.4	11.1	12.0
RIL2-9	−1734.6	−32.2	−20.5	92.7	93.4	20.5	−38.0	9.2	9.3	28.5	37.0	34.3	25.6	32.7	12.9	23.0
RIL2-10	−1416.3	−37.9	−29.8	90.9	91.2	14.7	−26.5	0.6	10.1	23.3	19.8	44.4	17.1	29.7	32.0	−4.7
RIL2-11	−1948.3	−75.8	−35.4	91.4	93.4	17.4	−27.8	3.9	−12.8	24.9	18.6	38.7	19.8	25.3	22.4	3.7

The abbreviations of Na^+^, K^+^, Ca^2+^, RWC, CT, Fv/Fm, Chla/Chlb, Chlt, PDW, GLA, GNPS, GY, BY, and HI indicate content of sodium, potassium, and calcium (mmol kg^−1^ DW), relative water content (%), canopy temperature (°C), maximum quantum PSII photochemical efficiency, chlorophyll a/b ratio, total chlorophyll content (mg g^−1^ FW), plant dry weight (g plant^−1^), green leaf area (cm^2^ plant^−1^), grain number per spike, biological yield (kg m^2^), grain yield (kg m^2^), and harvest index (%), respectively. The color gradient, which shifts from dark blue to dark orange, represents the varying reduction percentages of the trait as it smoothly transitions from the lowest to the highest values.

**Table 3:** Mean values of different agro-physiological traits for each group, identified based on the performance of genotypes under control and salinity stress separately or based on stress tolerance indices (STIs).

	Control Condition			Salinity Stress Condition			STIs		
Groups	Group 1	Group 2	Group 3	Group 1	Group 2	Group 3	Group 1	Group 2	Group 3
Grouping genotypes based on ionic traits									
Genotypes No.	2	3	17 (S61 + S93 + S1)	10	6 (S61 + S1)	6 S93	10 (S61+ S93 + S1)	6	6
Na^+^	136.86	100.55	90.20	1554.58	2130.78	1890.00	1072.17	822.00	872.66
K^+^	1132.62	747.51	1040.88	1169.08	807.37	975.65	988.13	1128.60	940.49
Ca^2+^	468.47	415.38	448.91	546.06	465.38	505.46	468.13	504.53	473.51
K^+^/Na^+^	8.27	7.49	11.58	0.76	0.38	0.52	5.77	6.21	4.92
Ca^2+^/Na^+^	3.42	4.14	5.05	0.36	0.22	0.27	2.39	2.61	2.71
Grouping genotypes based on physiological traits									
Genotypes No.	10	2	10 (S93 + S1 + S61)	9 (S61)	3 (S93)	10 (S1)	4	3 (S61)	15 (S93 + S1)
RWC	81.92	80.12	79.25	64.35	69.33	66.93	75.57	71.68	73.12
CT	26.47	26.48	27.79	38.08	34.55	35.62	30.33	32.70	31.96
Fv/Fm	0.82	0.79	0.81	0.76	0.82	0.78	0.81	0.78	0.79
Chla/Chlb	2.93	2.39	2.37	1.86	2.64	2.43	2.35	2.94	2.34
Chlt	4.31	4.42	4.04	2.66	3.56	3.18	4.17	3.09	3.56
Grouping genotypes based on growth and yield traits									
Genotypes No.	8	3 (S 93)	11 (S61 + S1)	10	3 (S61)	9 (S93 + S1)	15 (S93 + S1 + S61)	1	6
PDW	6.59	5.43	5.50	4.71	3.51	4.19	4.90	6.06	5.48
GLA	291.01	195.01	199.90	150.31	117.23	135.03	173.04	218.73	212.79
GNPS	53.17	50.48	48.93	41.32	35.00	37.74	43.62	48.16	47.33
GY	648.28	557.76	530.83	416.76	330.18	365.54	460.34	554.59	518.95
BY	2013.09	1845.35	1583.43	1462.93	979.76	1347.54	1488.59	1565.39	1747.19
HI	32.41	30.34	33.73	28.87	34.12	27.66	30.19	36.09	29.47

The abbreviations of Na^+^, K^+^, Ca^2+^, RWC, CT, Fv/Fm, Chla/Chlb, Chlt, PDW, GLA, GNPS, GY, BY, and HI indicate content of sodium, potassium, and calcium (mmol kg^−1^ DW), relative water content (%), canopy temperature (°C), maximum quantum PSII photochemical efficiency, chlorophyll a/b ratio, total chlorophyll content (mg g^−1^ FW), plant dry weight (g plant^−1^), green leaf area (cm^2^ plant^−1^), grain number per spike, biological yield (kg m^2^), grain yield (kg m^2^), and harvest index (%), respectively. S93, S61, and S1 are the names of three parents: Sakha 93, Sakha 61, and Sids 1, respectively.

**Table 4:** Full name, abbreviations (Abb.), formula, and references (Ref.) of each stress tolerance index (STI) calculated in this study.

Full Index Names	Abb.	Formula	Ref.
Stress Tolerance Index	STI	(V_C_ × V_S_)/(V_Ć_)^2^	[33]
Yield Stability Index	YSI	V_S_/V_C_	[60]
Mean Productivity Index	MPI	(V_C_ + V_S_)/2	[61]
Relative Efficiency Index	REI	(V_S_/V_Ś_) × (V_C_/V_Ć_)	[62]

Where V_C_ and V_S_ represent the trait values for each genotype under control and salinity stress conditions, respectively. V_Ć_ and V_Ś_ represent the mean trait values for all genotypes under control and salinity stress conditions, respectively.

## Data Availability

All data are presented within the article.

## Supplementary Materials

The following supporting information can be downloaded at https://www.mdpi.com/article/10.3390/plants14162488/s1, Figure S1: The variation in ion contents and their ratios among genotypes under control and salinity stress conditions; Figure S2: The variation in physiological traits among genotypes under control and salinity stress conditions; Figure S3: The variation in growth and yield traits among genotypes under control and salinity stress conditions.

## References

[1] Shiferaw B., Smale M., Braun H.J., Duveiller E., Reynolds M., Muricho G. (2013). Crops that feed the world 10. Past successes and future challenges to the role played by wheat in global food security. Food Secur..

[2] El-Hendawy S.E., Hassan W.M., Al-Suhaibani N.A., Refay Y., Abdella K.A. (2017). Comparative performance of multivariable agro-physiological parameters for detecting salt tolerance of wheat cultivars under simulated saline field growing conditions. Front. Plant Sci..

[3] Dadshani S., Sharma R.C., Baum M., Ogbonnaya F.C., Léon J., Ballvora A. (2019). Multi-dimensional evaluation of response to salt stress in wheat. PLoS ONE.

[4] Jeyasri R., Muthuramalingam P., Satish L., Pandian S., Chen J.-T., Ahmar S., Wang X., Mora-Poblete F., Ramesh M. (2021). An overview of abiotic stress in cereal crops: Negative impacts, regulation, biotechnology and integrated omics. Plants.

[5] Munns R., James R.A., Läuchli A. (2006). Approaches to increasing the salt tolerance of wheat and other cereals. J. Exp. Bot..

[6] Tavakkoli E., Rengasamyand P., Mcdonald G.K. (2010). The response of barley to salinity stress differs between hydroponics and soil systems. Funct. Plant Biol..

[7] Oyiga B.C., Sharma R.C., Shen J., Baum M., Ogbonnaya F.C., Leon J., Ballvora A. (2016). Identification and characterization of salt tolerance of wheat germplasm using a multivariable screening approach. J. Agron. Crop Sci..

[8] Moustafa E.S.A., Ali M.M.A., Kamara M.M., Awad M.F., Hassanin A.A., Mansour E. (2021). Field sreening of wheat advanced lines for salinity tolerance. Agronomy.

[9] Pongprayoon W., Tisarum R., Theerawittaya C., Cha-Um S. (2019). Evaluation and clustering on salt-tolerant ability in rice genotypes (*Oryza sativa* L. subsp. indica) using multivariate physiological indices. Physiol. Mol. Biol. Plants.

[10] Mubushar M., El-Hendawy S., Tahir M.U., Alotaibi M., Mohammed N., Refay Y., Tola E. (2022). Assessing the suitability of multivariate analysis for stress tolerance indices, biomass, and grain yield for detecting salt tolerance in advanced spring wheat lines irrigated with saline water under field conditions. Agronomy.

[11] Matkovíc Stojšin M., Petrovíc S., Banjac B., Zěcevíc V., Roljevíc Nikolíc S., Majstorovíc H., Ðordevíc R., Kneževíc D. (2022). Assessment of genotype stress tolerance as an effective way to sustain wheat production under salinity stress conditions. Sustainability.

[12] Tahir M.U., El-Hendawy S., Al-Suhaibani N. (2024). Comparative performance of ionic and agrophysiological traits for detecting salt tolerance in wheat genotypes grown in real field conditions. Life.

[13] Tao R., Ding J., Li C., Zhu X., Guo W., Zhu M. (2021). Evaluating and screening of agro-physiological indices for salinity stress tolerance in wheat at the seedling stage. Front. Plant Sci..

[14] Chaurasia S., Kumar A., Singh A.K. (2022). Comprehensive evaluation of morpho-physiological and ionic traits in wheat (*Triticum aestivum* L.) genotypes under salinity stress. Agriculture.

[15] Akram M., Alia Q., Alib S., El-Sheikhd M.A., Sarker P.K. (2025). Selection of salt-tolerant wheat genotypes for better yield considering physiobiochemical attributes and antioxidative defence potential: Agronomic traits and stress tolerance indices. Acta Agric. Scand. Sect. B-Soil Plant Sci..

[16] Saddiq M.S., Iqbal S., Hafeez M.B., Ibrahim A.M., Raza A., Fatima E.M., Baloch H., Woodrow P., Ciarmiello L.F. (2021). Effect of salinity stress on physiological changes in winter and spring wheat. Agronomy.

[17] Irshad A., Ahmed R.I., Ur Rehman S., Sun G., Ahmad F., Sher M.A., Aslam M.Z., Hassan M.M., Qari S.H., Aziz M.K. (2022). Characterization of salt tolerant wheat genotypes by using morpho-physiological, biochemical, and molecular analysis. Front. Plant Sci..

[18] Orzechowska A., Trtílek M., Tokarz K.M., Szymańska R., Niewiadomska E., Rozpądek P., Wątor K. (2021). Thermal analysis of stomatal response under salinity and high light. Int. J. Mol. Sci..

[19] Aloui M., Mahjoub A., Cheikh N.B., Ludidi N., Abdelly C., Badri M. (2022). Genetic variation in responses to salt stress in tunisian populations of *Medicago ciliaris*. Agronomy.

[20] Guidi L., Lo Piccolo E., Landi M. (2019). Chlorophyll fluorescence, photoinhibition and abiotic stress: Does it make any difference the fact to Be a C3 or C4 species? Front. Plant Sci..

[21] Stefanov M.A., Rashkov G.D., Apostolova E.L. (2022). Assessment of the Photosynthetic apparatus functions by chlorophyll fluorescence and P700 absorbance in C_3_ and C_4_ plants under physiological conditions and under salt stress. Int. J. Mol. Sci..

[22] Aizpour K., Shakiba M.R., Sima N.A.K.K., Alyari H., Mogaddam M., Esfandiari E., Pessarakli M. (2010). Physiological response of spring durum wheat genotypes to salinity. J. Plant Nutr..

[23] Pour-Aboughadareh A., Mehrvar M.R., Sanjani S., Amini A., Nikkhah-Chamanabad H., Asadi A. (2021). Effects of salinity stress on seedling biomass, physiochemical properties, and grain yield in different breeding wheat genotypes. Acta Physiol. Plant..

[24] Xu Y., Bu W., Xu Y., Fei H., Zhu Y., Ahmad I., Nimir N.E.A., Zhou G., Zhu G. (2024). Effects of salt stress on physiological and agronomic traits of rice genotypes with contrasting salt tolerance. Plants.

[25] Munns R., Rebetzke G.J., Husain S., James R.A., Hare R.A. (2003). Genetic control of sodium exclusion in durum wheat. Aust. J. Agric. Res..

[26] Jiang C., Zu C., Lu D., Zheng Q., Shen J., Wang H., Li D. (2017). Effect of exogenous selenium supply on photosynthesis, Na^+^ accumulation and antioxidative capacity of maize (*Zea mays* L.) under salinity stress. Sci. Rep..

[27] Hnilickova H., Kraus K., Vachova P., Hnilicka F. (2021). Salinity stress affects photosynthesis, malondialdehyde formation, and proline content in *Portulaca oleracea* L. *Plants*
**2021**, *10*, 845. Plants.

[28] El-Hendawy S.E., Hu Y., Schmidhalter U. (2005). Growth, ion content, gas exchange, and water relations of wheat genotypes differing in salt tolerances. Aust. J. Agric. Res..

[29] Gharib M., Qabil N., Salem A., Ali M., Awaad H., Mansour E. (2020). Characterization of wheat landraces and commercial cultivars based on morpho-phenological and agronomic traits. Cereal Res. Commun..

[30] Mansour E., Moustafa E.S., Desoky E.-S.M., Ali M.M., Yasin M.A., Attia A., Alsuhaibani N., Tahir M.U., El-Hendawy S. (2020). Multidimensional evaluation for detecting salt tolerance of bread wheat genotypes under actual saline field growing conditions. Plants.

[31] Hussain S., Hussain S., Ali B., Ren X., Chen X., Li Q., Ahmad N. (2021). Recent progress in understanding salinity tolerance in plants: Story of Na^+^/K^+^ balance and beyond. Plant Physiol. Biochem..

[32] Singh S., Sengar R.S., Kulshreshtha N., Datta D., Tomar R.S., Rao V.P., Garg D., Ojha A. (2015). Assessment of multiple tolerance indices for salinity stress in bread wheat (*Triticum aestivum* L.). J. Agric. Sci..

[33] Fernandez G.C.J. Effective selection criteria for assessing plant stress tolerance. Proceedings of the International Symposium on Adaptation of Vegetables and Other Food Crops in Temperature and Water Stress.

[34] Khalili M., Pour-Aboughadareh A., Naghavi M.R. (2016). Assessment of drought tolerance in barley: Integrated selection criterion and drought tolerance indices. Environ. Exp. Biol..

[35] Hammam K.A., Negim O. (2014). Evaluation of wheat genotypes and some soil properties under saline water irrigation. Ann. Agric. Sci..

[36] Mubushar M., El-Hendawy S., Dewir Y.H., Al-Suhaibani N. (2024). Ability of different growth indicators to detect salt tolerance of advanced spring wheat lines grown in real field conditions. Plants.

[37] El-Hendawy S., Ruan Y., Hu Y., Schmidhalter U. (2009). A comparison of screening criteria for salt tolerance in wheat under field and controlled environmental conditions. J. Agron. Crop Sci..

[38] Kotula L., Zahra N., Farooq M., Shabala S., Siddique K.H. (2024). Making wheat salt tolerant: What is missing?. Crop J..

[39] Quan X., Liang X., Li H., Xie C., He W., Qin Y. (2021). Identification and characterization of wheat germplasm for salt tolerance. Plants.

[40] Isayenkov S.V., Maathuis F.J.M. (2019). Plant salinity stress: Many unanswered questions remain. Front. Plant Sci..

[41] Xue Z.Y., Zhi D.Y., Xue G.P., Zhang H., Zhao Y.X., Xia G.M. (2004). Enhanced salt tolerance of transgenic wheat (*Tritivum aestivum* L.) expressing a vacuolar Na^+^/H^+^ antiporter gene with improved grain yields in saline soils in the field and a reduced level of leaf Na^+^. Plant Sci..

[42] Wu H. (2018). Plant salt tolerance and Na^+^ sensing and transport. Crop J..

[43] Traye I.D., Oli N.M., Weng X., Li K., Suliman M.S.E., Guo X., Zhou G., Zhu G., Xu Y. (2025). Salinity tolerance in wheat: Mechanisms and breeding approaches. Plants.

[44] Zhu J., Fan Y., Shabala S., Li C., Lv C., Guo B., Xu R., Zhou M. (2020). Understanding mechanisms of salinity tolerance in barley by proteomic and biochemical analysis of near-isogenic lines. Int. J. Mol. Sci..

[45] Boussora F., Triki T., Bennani L., Bagues M., Ali S.B., Ferchichi A., Ngaz K., Guasmi F. (2024). Mineral accumulation, relative water content and gas exchange are the main physiological regulating mechanisms to cope with salt stress in barley. Sci. Rep..

[46] Arfan M., Athar H.R., Ashraf M. (2007). Does exogenous application of salicylic acid through the rooting medium modulate growth and photosynthetic capacity in two differently adapted spring wheat cultivars under salt stress?. J. Plant Physiol..

[47] Li Y., Liu G.B., Gao H.W., Sun G.Z., Zhao H.M., Xie N. (2010). A comprehensive evaluation of salt-tolerance and the physiological response of *Medicago sativa* at the seedling stage. Acta Pratacult. Sin..

[48] Ma Y., Wei Z., Liu J., Liu X., Liu F. (2021). Growth and physiological responses of cotton plants to salt stress. J. Agron. Crop Sci..

[49] Sharma J.K., Sihmar M., Santal A.R., Singh N.P. (2021). Physiological and biochemical responses of seedlings of six contrasting barley (*Hordeum vulgare* L.) cultivars grown under salt-stressed conditions. J. Appl. Nat. Sci..

[50] Chen S., Feng J., Xiong Y., Xiong Y., Liu Y., Zhao J., Dong Z., Ma X., Yan L. (2023). Evaluation and screening of wild *Elymus sibiricus* L. germplasm resources under salt stress. Agronomy.

[51] Munns R., Passioura J.B., Colmer T.D., Byrt C.S. (2020). Osmotic adjustment and energy limitations to plant growth in saline soil. New Phytol..

[52] Yadav T., Kumar A., Yadav R.K., Yadav G., Kumar R., Kushwaha M. (2020). Salicylic acid and thiourea mitigate the salinity and drought stress on physiological traits governing yield in pearl millet–wheat. Saudi J. Biol. Sci..

[53] Soni S., Kumar A., Sehrawat N., Kumar A., Kumar N., Lata C., Mann A. (2021). Effect of saline irrigation on plant water traits, photosynthesis and ionic balance in durum wheat genotypes. Saudi J. Biol. Sci..

[54] Feng K., Licao C., Shuzuo L., Jianxin B., Wang M., Song W., Xiaojun N. (2018). Comprehensive evaluating of wild and cultivated emmer wheat (*Triticum turgidum* L.) genotypes response to salt stress. Plant Growth Regul..

[55] Genc Y., Taylor J., Lyons G., Li Y., Cheong J., Appelbee A., Oldach K., Sutton T. (2019). Bread wheat with high salinity and sodicity tolerance. Front. Plant Sci..

[56] Saqib Z.A., Akhtar J., Ul-Haq M.A., Ahmad I., Bakhat H.F. (2012). Rationality of using various physiological and yield related traits in determining salt tolerance in wheat. Afr. J. Biotechnol..

[57] El-Hendawy S.E., Hu Y., Yakout G.M., Awad A.M., Hafiz S.E., Schmidhalter U. (2005). Evaluating salt tolerance of wheat genotypes using multiple parameters. Eur. J. Agron..

[58] Lichtenthaler H.K., Wellburn A.R. (1983). Determinations of total carotenoids and chlorophylls A and B of leaf extracts in different solvents. Biochem. Soc. Trans..

[59] Jones H.G. (2004). Application of thermal imaging and infrared sensing in plant physiology and eco-physiology. Adv. Bot. Res..

[60] Bouslama M., Schapaugh W.T. (1984). Stress tolerance in soybean. Part 1: Evaluation of three screening techniques for heat and drought tolerance. Crop. Sci..

[61] Rosielle A., Hamblin J. (1981). Theoretical aspects of selection for yield in stress and non-stress environment. Crop Sci..

[62] Fischer R., Maurer R. (1978). Drought resistance in spring wheat cultivars. I. Grain yield responses. Aust. J. Agric. Res..

