# Supplementary material for: Evaluating the Necessity of a Control Treatment for Assessing Salt Tolerance in Wheat Genotypes Based on Agro-Physiological Traits in Real-Field Conditions
Source: Plants (Basel). 2025 Aug 11;14(16):2488. doi: 10.3390/plants14162488 (PMC12388888; doi:10.3390/plants14162488)
Supplement: Supplementary file 1 [file plants-14-02488-s001.zip › plants-3775490-supplementary.pdf]

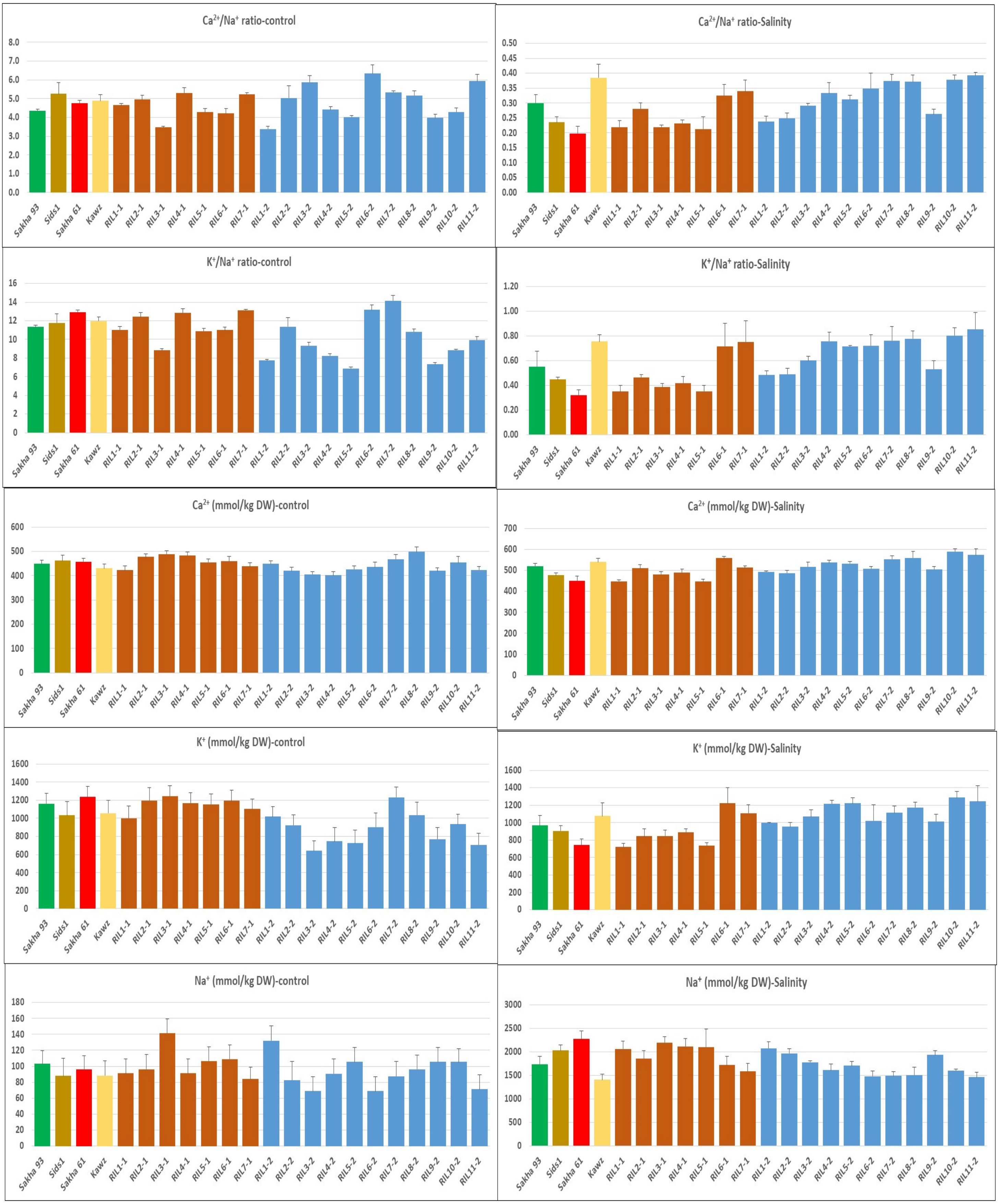

Figure S1. The variation of ion contents and their ratio among genotypes under control and salinity stress conditions. Salt-tolerant genotypes were represented by green, salt-sensitive genotypes by red, RIL1 group by dark orange, and RIL2 group by blue colors. (means ± SE; n = 3)

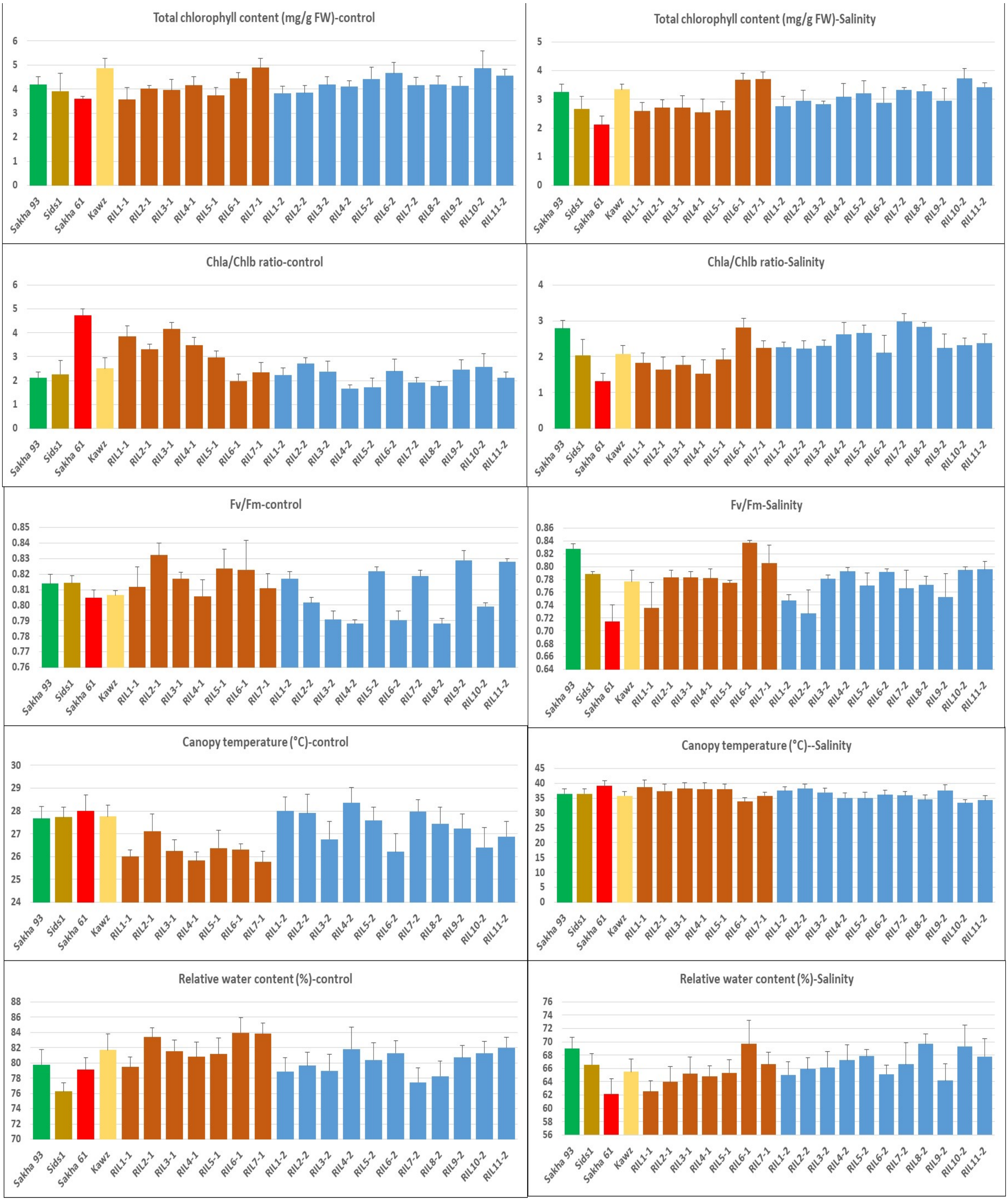

Figure S2. The variation of physiological traits among genotypes under control and salinity stress conditions. Salt-tolerant genotypes were represented by green, salt-sensitive genotypes by red, RIL1 group by dark orange, and RIL2 group by blue colors. (means ± SE; n = 3)

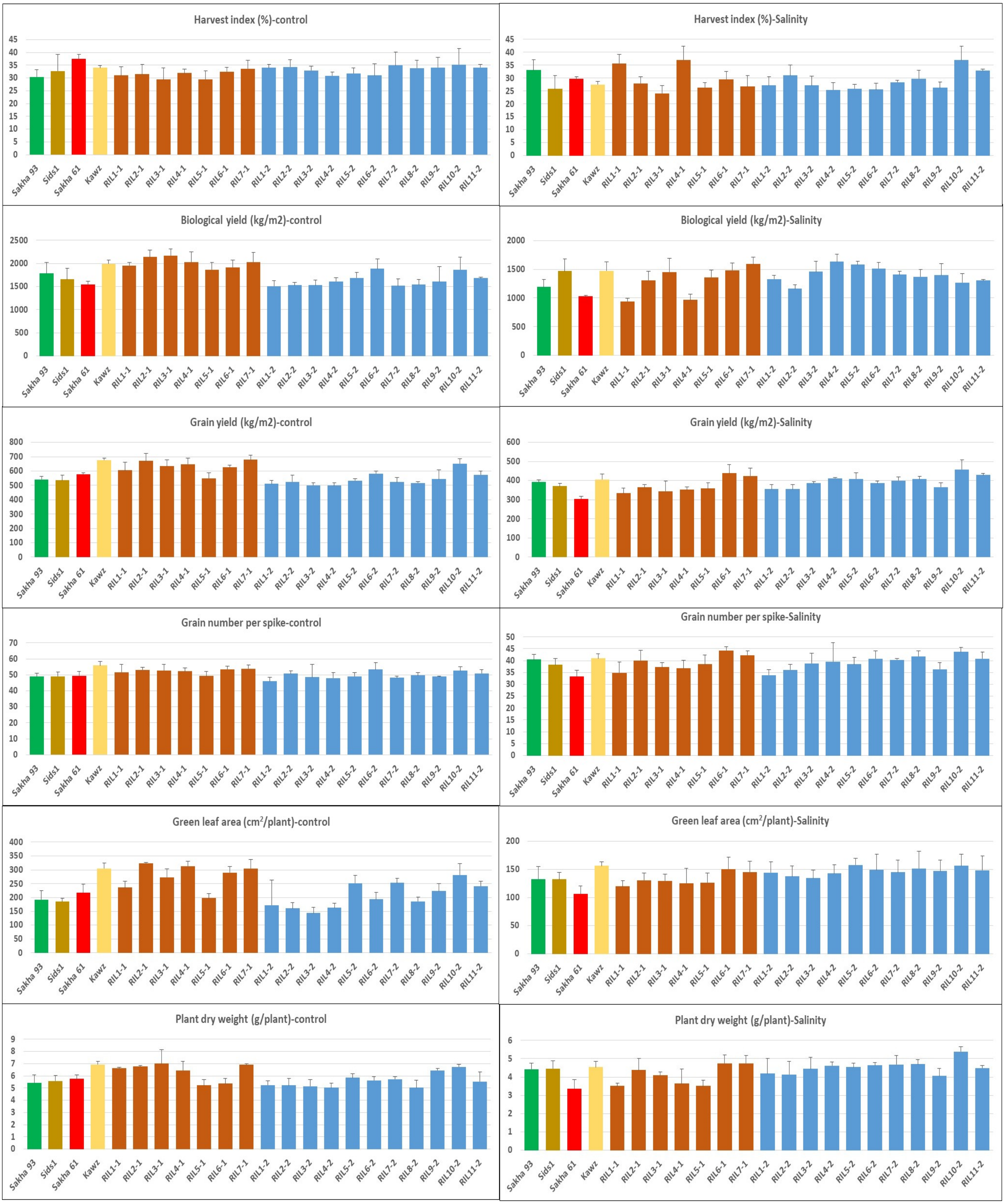

Figure S3. The variation of growth and yield traits among genotypes under control and salinity stress conditions. Salt-tolerant genotypes were represented by green, salt-sensitive genotypes by red, RIL1 group by dark orange, and RIL2 group by blue colors. (means ± SE; n = 3)
